# Pilot implementation of two specific problem lists before and after solid organ transplantation into routine care

**DOI:** 10.3389/fpsyg.2024.1481643

**Published:** 2025-01-17

**Authors:** Sanna Higgen, Evamaria Müller, Markus J. Barten, Doreen Eickhoff, Florian Grahammer, Martin Härter, Sabine Bart, Martina R. Sterneck, Angela Buchholz

**Affiliations:** ^1^Department of Medical Psychology, University Medical Center Hamburg-Eppendorf, Hamburg, Germany; ^2^Department of Cardiovascular Surgery, University Medical Center Hamburg-Eppendorf, Hamburg, Germany; ^3^University Transplant Center, University Medical Center Hamburg- Eppendorf, Hamburg, Germany; ^4^Center for Internal Medicine, University Medical Center Hamburg-Eppendorf, Hamburg, Germany; ^5^Department Health Sciences, University of Applied Sciences Hamburg, Hamburg, Germany; ^6^Department of Internal Medicine, University Medical Center Hamburg- Eppendorf, Hamburg, Germany

**Keywords:** transplant recipients, organ transplantation, psychological distress, implementation research, symptom assessment

## Abstract

**Introduction:**

Psychosocial distress and mental health problems are common in patients before and after solid organ transplantation and can negatively affect morbidity, mortality, and adherence. Even though regular screening is recommended to identify patients with high levels of distress, the implementation in routine care has been insufficient so far.

**Methods:**

Two newly developed problem lists for patients before and after transplantations were pilot implemented for 8 weeks at the Medical Center Hamburg Eppendorf (UKE) to identify factors facilitating and impeding their implementation.

**Results:**

Health care professionals evaluated its appropriateness, feasibility, and the cooperation with the psychologists before (HCPs: *n* = 23) and after (HCPs: *n* = 19) the implementation. Four psychologists assessed the appropriateness and feasibility by answering to open-ended and close-ended questions. Additionally, patients before (*n* = 8) and after (*n* = 100) transplantation filled out the screening and rated its acceptance. Only the data of the patients after transplantation were analyzed due to the small sample size of patients before transplantation. HCPs and psychologists rated the screenings as very appropriate [HCPs: *M* = 3.84 (SD = 0.77) to *M* = 4.32 (SD = 0.58)]. It was also highly accepted among patients [*M* = 4.23 (SD = 0.85) to *M* = 4.68 (SD = 0.65)]. Contentment with the psychological support and understanding of the mental health problems among HCPs increased significantly from before to after the implementation (*U* = 107.50, *p* < 0.05, *r* = 0.33; *U* = 107.00, *p* < 0.05, *r* = 0.34). The feasibility of the problem list post-Tx in routine care, however, was seen as challenging [HCPs: *M* = 3.11 (SD = 1.05) to *M* = 3.47 (SD = 1.07)].

**Discussion:**

The distress screening was accepted and improved the cooperation between different professions. Barriers to implementation can be lack of staff and resources. Future studies should assess the adoption and sustainability of the screening in routine care.

## Introduction

For patients with end stage organ disease, transplantation often remains the only treatment that can save their lives (Lange and von der Lippe, 2009). While overall quality of life does improve after the transplantation, improvements in psychosocial functioning are less distinctive than in physical health (Dew et al., 2000; Schulz and Kroencke, 2015).

Patients encounter a variety of challenges during the entire transplantation process including evaluation before the transplantation, staying on the waiting list, surgery, and aftercare. Before transplantation patients might reduce social contacts in order to prevent infections or because they feel to impose a burden on relatives and friends (Ivarsson et al., 2011). The disease and treatment cause pain and distress (Li et al., 2012) while the waiting time for an organ can increase fear of death (Kuntz et al., 2015). After the transplantation patients can feel burdened by the realization that their health is still limited and that they will have to take immunosuppressants for the rest of their life (Köllner and Archonti, 2003). Besides, fear of organ loss or infection are common (Baranyi et al., 2013).

The rate of mental disorders is markedly increased in transplant patients compared to the general population (Rosenberger et al., 2012; Evans et al., 2015). The prevalence of symptoms changes depending on the time since transplantation (Annema et al., 2015). Symptoms of anxiety and depression were more prevalent in the first 2 years and in the long term (15 years) after transplantation. Symptoms of PTS were more prevalent in the first 5 years after transplantation. In total, up to 60% of solid organ recipients suffer from affective disorders which are associated with higher morbidity and mortality (Dew and DiMartini, 2005; Heinrich and Marcangelo, 2009; Rosenberger et al., 2012; Corbett et al., 2013; Rogal et al., 2013; Dew et al., 2015; Smith et al., 2016). Some transplant recipients also show symptoms of anxiety and post-traumatic stress (Annema et al., 2015). The psychological distress impairs the quality of life of patients before and after transplantation (Heinrich and Marcangelo, 2009; Baranyi et al., 2013; Novak et al., 2013) and can negatively impact their adherence (Achille et al., 2006; Heinrich and Marcangelo, 2009). Nonadherence in turn can increase the rate of graft loss (Butler et al., 2004).

However, despite the considerable impact of transplantation on patients’ quality of life health care professionals (HCPs) generally pay less attention to psychological than physical symptoms and patients with symptoms of distress often remain unrecognized and underserved (Mehnert et al., 2006). In order to detect patients suffering from psychological distress regular screening is highly recommended (Heinrich and Marcangelo, 2009; DiMartini et al., 2011; Rosenberger et al., 2012; Corbett et al., 2013; Miller et al., 2013; Dew et al., 2015). Screening was shown to be especially beneficial when it is applied to more vulnerable, high-risk patients and when the implementation is supported by ongoing staff training or supervision (Mitchell et al., 2012). Screening can improve communication between patients and clinicians and increase the detection and diagnosis of mental disorders (Mitchell et al., 2012). The relevance of psychosocial care in the context of solid organ transplantation has recently been summarized in a clinical practice guideline including clear recommendations for psychosocial screening and multiprofessional care (de Zwaan et al., 2023).

Despite the apparent advantages of employing screenings in routine care (Mitchell et al., 2012) the implementation of screenings is challenging, oftentimes leading to the eventual cessation of its use (Pirl et al., 2007; Mitchell et al., 2008; Dudgeon et al., 2012). Lack of staff, competing demands and staff turn-over are common barriers to the implementation (Dudgeon et al., 2012; Mitchell et al., 2012; Knies et al., 2019). Acceptance and institutional support in implementing the screening, however, can facilitate the application (Mitchell et al., 2012; Knies et al., 2019). Piloting the screening in an implementation study can help identify possible obstacles and enable the integration into routine care. Important implementation outcomes are acceptability, adoption, appropriateness, feasibility, fidelity, implementation cost, penetration, and sustainability (Proctor et al., 2011).

Although there are many screening tools available primarily covering symptoms of anxiety and depression, there has been no screening for psychosocial distress specific to patients before or after solid organ transplantation. As it is essential that an assessment of distress also includes psychosocial problems most relevant to the specific patient group (Brennan et al., 2012). Müller et al. (under review)^1^ recently developed two specific problem lists reflecting the concerns of patients before and after transplantation, respectively. Both lists can be added to a short general measure of distress, i.e., the NCNN Distress Thermometer (Donovan et al., 2014) and used in routine care. Aim of the screening is the early identification of patients suffering from psychosocial distress and to provide suitable care.

Aim of this subsequent study was to identify barriers and facilitators for the implementation of the problem lists as screening tool in an university transplantation center. We assessed acceptability, appropriateness and feasibility as these constitute the most important outcomes when implementing a new intervention (Peters et al., 2013).

## Materials and methods

### Study design and ethical approval

The study used an observational design without control group. We surveyed HCPs before and after a pilot implementation phase of 8 weeks at one inpatient and one outpatient clinic being part of the University Transplant Center (UTC). Patients were included in the study during the implementation phase. The study was carried out in accordance with the Code of Ethics of the Declaration of Helsinki and was approved by the Local Ethics Committee of the Center for Psychosocial Medicine, University Medical Center Hamburg (UKE), Germany (registration code LPEK-0029).

### Setting and sample

This pilot study was conducted at the University Medical Center Hamburg-Eppendorf in the outpatient clinic for heart failure, heart and lung transplantation and artificial heart systems and on the transplant ward for visceral transplant surgery of the UTC. HCPs working with transplant patients including physicians, nurses, and medical assistants were eligible to participate in the study. Before the implementation, 23 HCPs participated. After the implementation 19 HCPs participated in the study, 4 of which worked as transplant psychologists. The transplant psychologists are part of the health care team at the included in- and outpatient clinics. Additionally, patients who were wait-listed for a transplantation or had received an organ and were currently being treated in one of the participating wards / outpatient clinics were eligible to participate. In total 111 patients participated in the study.

### Measures

#### HCPs

HCPs were asked to indicate sociodemographic data (age, gender, position, years of work experience, current work with patients before or after transplantation). To assess the *appropriateness* of the problem list, the items concerning “relevance” (Bartholomew et al., 2007) of the TCU Workshop Evaluation Form WEVAL (Institute of Behavioral Research, 2002) were translated using the TRAP-D approach (Harkness et al., 2004) and adapted to the problem list. To evaluate *feasibility*, the items concerning “program support” (Bartholomew et al., 2007) were translated and adapted to the problem list analogous to the procedure for appropriateness. Five self-developed items regarding the cooperation with the transplant psychologists were asked before and after the implementation of the screening. All items were rated on a 5-point Likert-scale. Participating psychologists were asked to respond to seven additional self-developed statements, because they were considered as working most frequently with the screening. These additional items covered the *usage* and *usefulness* of the screening. Participants had the opportunity of adding a comment to each question. Also, three open-ended questions were asked on the expected advantages, challenges, and prerequisites for long-term use of the problem list.

#### Patients

Patients were asked to report a range of sociodemographic data such as age, gender, mother tongue, last educational institution graduated, employment, current sick leave, wait-listing for organ transplantation, type of organ transplanted (if applicable). In addition, patients replied to single questions on their general and mental health. Answers were given on a 5-point Likert-scale, ranging from 1 “bad” to 5 “excellent.” Furthermore, patients were asked to complete a distress screening consisting of four different parts:

1. The NCCN distress Thermometer (Donovan et al., 2014) as generic measure of psychological distress ranging from 0 to 10.
2. One of the newly developed problem lists according to the patients’ transplantation status (before or after transplantations, see supplementary file 1). The pre-transplantation list contains 21 and the post-transplantation list contains 22 items plus an open-response field for problems not included in the lists. The problems of both lists pertain to four categories: “problems in everyday-life,” “social problems,” “worries and fears” and “physical and mental problems.”
3. The short form of the Patient Health Questionnaire (PHQ-4) as a screening for depressive and anxiety symptoms (Kroenke et al., 2009). An overall sum-score for the PHQ-4 as well as sum-scores for the subscales PHQ-2 and GAD-2 can be generated to identify patients suffering from depression or anxiety. The PHQ-4-score is categorized as follows: normal (0–2), mild (3–5), moderate (6–8) and severe (9–12).
4. At the end of the screening patients can indicate whether they would like to talk to a psychologist.

The last part of the patient questionnaire assessed acceptance of the new measure.

Questions were developed following the *Acceptability E-Scale* by Tariman et al. (2011). Of the six original questions five were translated according to the TRAP-D approach (Harkness et al., 2004). The sixth item (“How helpful to you was this xy in describing your symptoms and QOL?”) was omitted because it did not fit the focus of the study. Questions were answered on a 5-point Likert-scale. Besides, patients had the opportunity to write a comment on the problem list at the end of the questionnaire. A result of 80% of the highest reachable score is considered to represent acceptance by the users (Tariman et al., 2011). This would be a total score of 20 points in this study.

### Procedure

HCPs filled out a questionnaire on the cooperation with the transplant psychologists before and after the implementation. Additionally, after the implementation HCPs as well as transplant psychologists were asked to rate the appropriateness and feasibility of the screening. Patients who agreed to participate received a consent form, the distress screening and a questionnaire regarding their acceptance of the screening from the study team. Patients that refused to participate were asked for their reasons to refuse. Inpatients received the screening during a regular visit from the transplant psychologists while recovering from transplantation. Outpatients were visiting the clinic for their regular check-ups before or after the transplantation. They received the screening from an HCP. The questionnaires were either sent to the study group via mail or were picked up by someone from the study team.

### Statistics

#### Quantitative

All data analyses were performed using IBM SPSS Statistics 27 (IBM Corporation, Amonk NY). Pairwise deletion was employed for missing data, therefore, sample sizes will differ depending on the scale (Lüdtke et al., 2007). Descriptive analyses on the sample were executed. Means and standard deviations of the items on the implementation outcome scale (appropriateness, feasibility) were calculated. To assess the rate of acceptance, the number of patients that rated the screening as acceptable (score ≥ 20) was calculated. To assess whether the cooperation with the transplant psychologists has improved non-parametric Mann–Whitney-U tests were performed with each single item. The effect size *r* was calculated for significant results. A significance level of *p* < 0.05 was set.

#### Qualitative

The comments patients made on the acceptance scale and the comments of the psychologists on the appropriateness and feasibility of the screening were summarized and sorted into categories, respectively.

## Results

### Sample characteristics

#### HCPs

Questionnaires were handed out to 47 HCPs before and after the implementation of the screening (35 inpatient and 12 outpatient HCPs). Before the implementation 23 HCPs participated, after the screening there were 19 respondents (Table 1).

**Table 1:** Clinical and demographic characteristics of participating health care professionals.

	Before implementation (t0)	After implementation (t1)
Total *n*	23	19
Age		
Mean (SD)	36.57 (9.85)	36.11 (7.89)
Range	22–59	23–52
Sex, *n* (%)		
Female	16 (69.9)	14 (73.7)
Male	7 (30.4)	5 (26.3)
Native language, *n* (%)	93	8
German	77 (82.8)	8 (100)
Other	16 (7.2)	-
Occupation, *n* (%)		
Nursing staff	9 (39.1)	2 (10.5)
Qualified medical employee	2 (8.7)	3 (15.8)
Social worker	-	-
Psychologist	-	4 (21.1)
Medical resident	7 (30.4)	7 (36.8)
Attending/head physician	5 (21.7)	2 (10.5)
Other	-	1 (5.3)
Professional experience with transplant patients, *n* (%)		
< 5 years	12 (52.2)	12 (63.2)
5–10 years	5 (21.7)	4 (21.1)
11–20 years	4 (17.4)	3 (15.8)
> 20 years	2 (8.7)	-
Clinical setting, *n* (%)		
Inpatient	15 (65.2)	9 (36.8)
Outpatient	8 (34.8)	10 (42.1)

#### Patients

In total 147 patients were asked to participate in the study of which 111 participated. As just eight patients were recruited before the transplantation only the results of the post-Tx patients will be presented (Table 2). Three patients were excluded because they gave no information on the organ that they received. 36 patients did not participate for various reasons such as lack of time, lack of German language skills, mistrust, no interest or because they did not meet the eligibility criteria.

**Table 2:** Clinical and demographic characteristics of the patient sample (*N* = 100).

	Post-Tx (*n* = 100)
Age, *n* (%)	98 (98)
Mean (SD)	54.3 (15.0)
Range	18–83
Sex, *n* (%)	100 (100)
Female	43 (43)
Male	57 (57)
Native language, *n* (%)	82 (82)
German	67 (81.7)
Other	15 (18.3)
Education, *n* (%)	100 (100)
Less than junior high school (< 10 y)	9 (9)
Junior high school (10y)	52 (52)
High school (12–13 y)	20 (20)
College/university	18 (18)
Other	1 (1)
Occupational status, *n* (%)	99 (99)
Working	32 (32.3)
Retired	53 (53.5)
Homemaker	1 (1)
Student	1 (1)
Unemployed	8 (8.1)
Other	4 (4)
Certified sick, *n* (%)	96 (96)
Yes	26 (27.1)
No	70 (72.9)
Clinical setting, *n* (%)	100 (100)
Inpatient	32 (32)
Outpatient	68 (68)
Organ (pre) transplant, *n* (%)	100 (100)
Lung	11 (11)
Heart	57 (57)
Kidney	14 (14)
Liver	18 (18)
Number of transplant operations, *n* (%) 1 2 3	90 (90) 85 (94.4) 4 (4.4) 1 (1.1)
General health*, *n* Mean (sd)	98 (98) 3.02 (0.90)
Mental health*, *n* Mean (sd)	99 (99) 3.18 (1.01)

Caption: *scale 1 (bad) to 5 (excellent).

### Implementation outcomes

#### Appropriateness and feasibility

The implementation outcomes as judged by the HCPs can be seen in Table 3. While the appropriateness has been rated almost completely with a mean score > 4, the feasibility of the instrument was rated in a mediocre range (> 3).

**Table 3:** Implementation outcomes from the HCPs’ perspective.

Question	*M* (SD)
Appropriateness (*n* = 19)	
Were you satisfied with the screening?	4.11 (0.46)
Would you feel comfortable using the screening?	4.26 (0.65)
The content of the screening is relevant for the needs of outpatients	4.32 (0.58)
Do you expect the screening to be used shortly?	3.84 (0.77)
Feasibility (*n* = 19)	
The clinic/outpatient centre has sufficient staff to implement the screening.	3.21 (1.03)
The clinic/outpatient centre has sufficient resources to implement the screening.	3.47 (1.07)
There is sufficient time to prepare the implementation of the screening.	3.11 (1.05)

Caption: scale 1 (I do not agree) to 5 (I agree completely).

The ratings of the seven statements by the psychologists were summarized to represent whether they agreed or disagreed/were uncertain (Table 4). The results demonstrate that psychologists agreed with the majority of the statements. Only the screenings’ usefulness for structuring the conversation and for providing feedback to HCPs was not approved.

**Table 4:** Appropriateness and feasibility of the screening from the psychologists’ perspective.

Statement	Disagree/ uncertain	Agree
The screening is useful for structuring the conversation with the patients.	3	1
The screening helps introducing topics that are otherwise rarely discussed.	1	3
The screening is helpful in providing feedback on the mental state of the patients to the physicians.	2	2
The screening is helpful in providing feedback on the mental state of the patients to the nurses.	2	2
The screening helps identifying distressed patients.	1	3
The screening helps identifying patients that need to talk.	1	3
The screening is helpful in organising appropriate on-going care for patients	1	3
Sum	3	17

Caption: number of psychologists that agreed or disagreed/were uncertain with the statements. Due to the small sample size, absolute numbers are presented.

The comments by the psychologists either highlighted *advantages* of the screening, alerted to *challenges* or were *suggestions* for improving the implementation of the screening (Table 5).

**Table 5:** Summary of comments made by the psychologists.

Examples	Quotation
Advantages	
Promote patients’ understanding of mental health Patients reflect on their own mental health Increase interdisciplinary cooperation No neglect of mental health Motive to offer a conversation to highly stressed patients (even if they indicate no need to talk) Identify distressed patients and relevant problem areas Involvement of patients in the treatment Facilitate a holistic treatment approach	“I see the benefit especially in identifying distressed patients, possibly improving support and the exchange with doctors and nurses.” (P3)
Challenges	
Implementation is time- and resource-demanding Difficulty understanding (language barriers, unclear wording) Reservations toward psychologists	“Additional workload for the different professions. Different acceptance of the screening or the transplant psychologists by patients. General reservations toward the discipline of psychology. Comprehensibility of the questions for patients - difficulties understanding, language barriers, etc.” (P2)
Suggestions	
Hand out the screening when patients first enter the clinic Ensure availability of results to all professions Clear distribution of tasks (handing out, analysis, discussion of results) with sufficient time Use for specific concerns rather than general mental health	“Integration in daily ward routine, but for that doctors, nurses and social service need to be interested in the information. That’s why I find it important to have the screening filled out at the beginning of the stay, when many things are still unclear and can be organized. The screening should be scanned and included in the patient record as information, if that was possible. Then everyone could see it, also social service, it might make it [the screening] more helpful” (P1)

#### Cooperation with psychological staff

**Figure 1:**
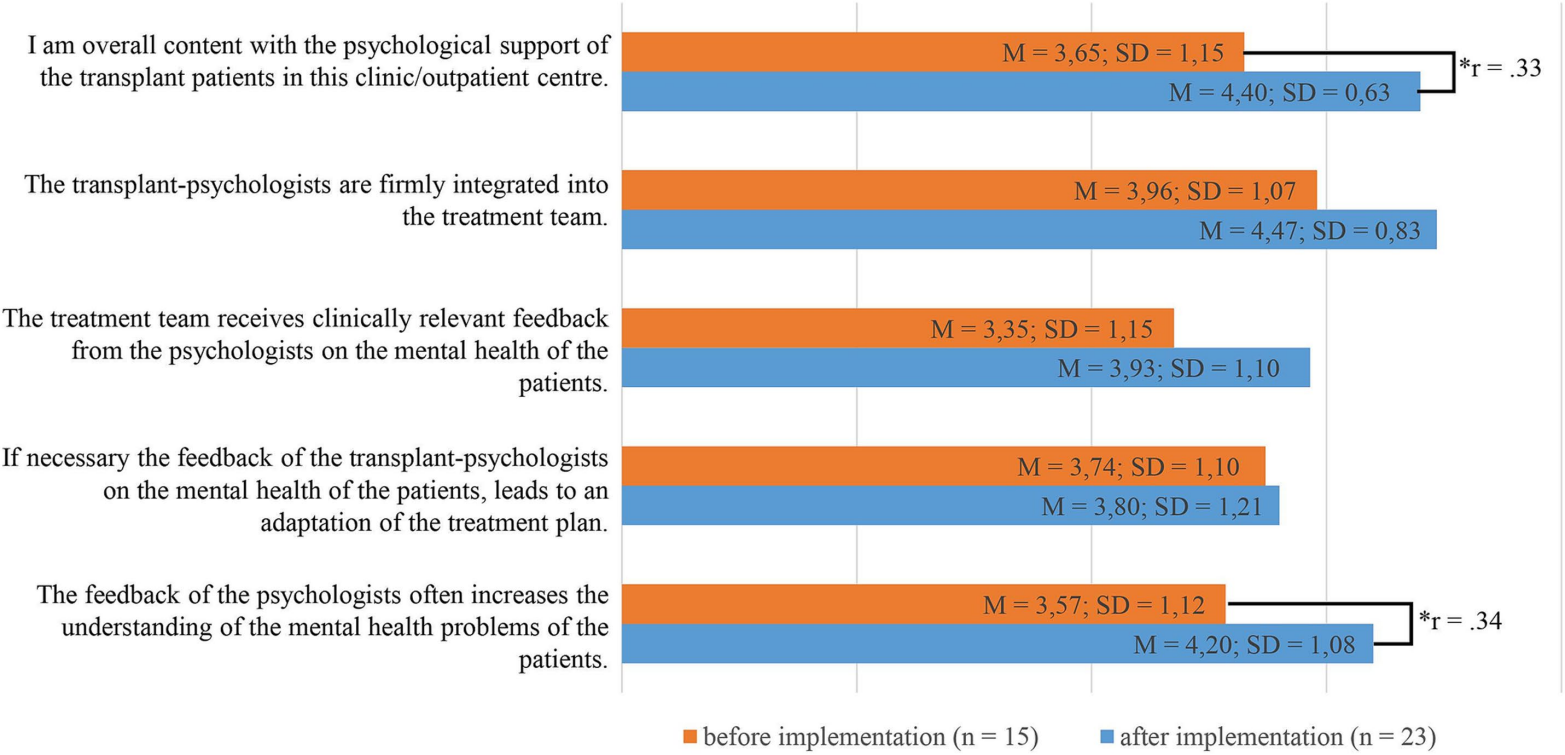
Cooperation with psychological staff before and after implementation of the screening: scale 1 (I do not agree) to 5 (I agree); * *p* < 0.05.

The cooperation with the psychologists improved significantly which was demonstrated in an increase in contentment with the care for patients (*U* = 107.5). Also, the HCPs’ understanding of the mental health problems of the patients improved through the feedback of the psychologists (Figure 1).

#### Acceptance

The average acceptance of the screening by the patients can be seen in Table 6.

**Table 6:** Acceptance of the screening by patients.

Question	*n*	*M* (SD)
How easy did you find the use of the screening?	100	4.55 (0.74)
How comprehensible were the question for you?	100	4.62 (0.71)
How much did you enjoy using the screening?	100	4.23 (0.85)
Was the required time for the screening acceptable?	100	4.68 (0.65)
How content are you overall with the screening?	99	4.36 (0.79)
Sum score	99	22.46 (2.72)

Caption: scale 1 (very difficult) to 5 (very easy).

The goal of an acceptance rate of 80% (sum score = 20) was achieved. 84 patients (84.8%) had a sum score of 20 or higher.

The answers to the open-ended questions were analyzed qualitatively. In total 18 patients commented on the screening which can be sorted into the five categories *problems in understanding*, *unsuitable items*, *unsuitable time frame*, *timing of the screening* and *positive reception of the screening* (Table 7).

**Table 7:** Summary of comments made by patients.

Examples	Quotation
Problems in understanding	
Language barriers Unclear wording Changing situation	“Some questions I could not clearly answer with yes or no because the situation keeps changing constantly.”
Unsuitable items	
Questions not suitable for inpatients No question on dealing with medication or behavior after Tx Questions too shallow	“Some questions were a bit difficult to answer as the hospital stay is a special situation while the questionnaire should actually represent the typical everyday life.
Unsuitable time frame	
One week insufficient for mood swings The first years are more meaningful	“Respresents only one week, the mood after HTx is often undulating.”
Timing of the screening	
Too late Too soon	“As I received my transplant 27 years ago I would have needed support sooner.”
Positive reception of the screening	
Considers psychological aspects Relevant items Helps dealing with the Tx	“I think it’s important to not only see the physical condition but also the psychological burden.”

## Discussion

This study tested the implementation of a distress screening including specific problem lists for transplant patients before and after the transplantation. As only eight patients participated before the transplantation these results were excluded from the analysis and only the results from patients after the transplantation were reported. The screening is accepted by HCPs and patients and is appropriate for the use with this patient group. It can improve the cooperation among HCPs and psychologists. However, the feasibility of the screening is perceived as average and patients, HCPs and psychologists see a range of challenges when implementing the screening in routine care.

The study helped to identify facilitators and barriers for the implementation of the screening. The outcomes show that HCPs considered the screening to be appropriate for the population of interest and felt comfortable using it. Psychologists also evaluated the screening to be appropriate and helpful. The screening can stimulate patients to reflect on their own mental health, facilitate the identification of distressed patients and improve the cooperation between professions. However, concerning the feasibility of the screening and the expected implementation participants were less certain. HCPs and psychologists identified lack of staff and resources as barriers to the implementation of the screening. Lack of staff or staff turn-over have previously been identified as impeding the implementation of screenings(Dudgeon et al., 2012; Mitchell et al., 2012; Knies et al., 2019). A clear distribution of tasks and timing management of the screening were suggested as solutions to these obstacles. The literature stresses the importance of institutional support and acceptance to facilitate the implementation of a screening (Mitchell et al., 2012; Knies et al., 2019). Other strategies that improve screening implementation are a formalized and uniform screening process, the formation of an interdisciplinary group that directs and evaluates the screening policy and a referral network to treat the distress (Ercolano et al., 2018).

The results on the patients’ acceptance scale demonstrate that the screening is highly accepted among patients. The screening was considered as comprehensible, enjoyable and convenient. Patients appreciated that their level of distress and psychosocial problems were taken into consideration and perceived the items as relevant. Yet, the timing of the screening and problems in understanding some items were named as potential barriers to the use of the screening. Language barriers and patient literacy were previously identified hindering screening implementation (Lo et al., 2016). However, in the content-valid development of the problem list, only German speaking patients could be reached (Müller et al., 2024). Adapting the problem list to different languages and cultures should be the next step to increase the inclusiveness and outreach of the screening.

The list was developed to be suitable for all types of organs. Yet, it would be interesting to see whether the items endorsed on the problem list differ depending on the organ transplanted. In this study, type of organ was likely confounded with the setting of the patients (in- vs. out-patients). Therefore, no additional analyses were done on the influence the transplanted organ might have on the results.

Even though psychologists were already a part of the clinical team before implementation of the screening, it had a positive impact on the collaboration between psychologists and the other HCPs. HCPs were more content with the psychological care of the patients and the feedback of the psychologist increased the HCPs’ understanding of the mental health problems of the patients. Improving the psychological care of patients is one central aim of implementing the distress screening.

This study is subject to some limitations. The sample of patients before the transplantation was very small, which is why the sample was excluded from the analyses. It is well known that patients on the waiting list are harder to reach compared with patients after transplantation. Before transplantation, the frequency of in- and outpatients hospital visits varies largely depending on the severity of the disease and the organ affected. It seems to be more difficult to identify convenient and meaningful time points for the application of the screening.

We did, however, expect to reach more patients on the waiting list in the outpatient clinic. It would be promising to use the pre-transplant problem list prior to the psychosocial evaluation. Due to the short study period this could not be realized in the current study. Furthermore, depending on the organ, not every patient undergoes psychosocial evaluation in Germany (de Zwaan et al., 2023). For those patients who are not undergoing regular psychosocial evaluation, the problem list could serve to identify patients in need and to refer to psychosocial evaluation.

Also, the sample of HCPs and psychologists is quite limited. Due to the small sample size results need to be interpreted with caution. The reasons for not participating were not gathered from the HCPs. It is likely that insufficient time and resources reduced the willingness of the staff to take part in the study.

Due to the anonymity of the study results could not be linked to clinical data. Therefore, patients that did not specify the organ they received had to be excluded as we could not be sure whether these patients really received a transplant or were accidentally included in the study.

As this was a pilot implementation no process data were gathered such as adoption in routine care and use of the screening by HCPs. Also, we did not determine the prevalence of mental disorders in our study sample and therefore cannot analyze associations of our distress screenings to diagnosed mental disorders. Future studies should assess the implementation of the screening and its relation to mental health orders.

Despite these limitations, this study demonstrates the appropriateness and acceptance of the newly developed distress screening for patients after transplantation. It ameliorates the cooperation between different professions and probably facilitates the early identification of patients with high levels of distress. Barriers to screening implementation were lack of staff and resources as well as inconvenient timing of the screening or problems in understanding. Factors that can facilitate and increase the use of the screening were acceptance of the screening, and an improved cooperation between professions. Patients appreciated that their psychosocial distress was considered. We recommend using the screening with the components employed in this study, i.e., the distress scale, the problem list and the PHQ-4. The feasibility is evaluated as challenging due to limitations of staff and resources. Before implementation, institutions should reflect on the right time and setting, and also on possible interventions following the screening, i.e., referral to a psychologist, discussing the issues raised in the problem list in the medical encounter or with the transplant nurse. Future studies should test the screening with a large sample of patients before transplantation. Besides, disparities between patients with different transplanted organs should be investigated. The adoption and sustainability of the screening when it is implemented in routine care should be assessed to identify factors that can increase the fidelity and penetration.

## Data Availability

The raw data supporting the conclusions of this article will be made available by the authors, without undue reservation.

## Footnotes

^1^ Müller, E, Härter, M., Higgen, S., Barten, M. J., Eickhoff, D., Grahammer, et al. (under review). The development and psychometric evaluation of specific problem lists reflecting psychosocial distress of patients before and after solid organ transplantation. *Front. Psychol.*
