# Supplementary material for: Pilot implementation of two specific problem lists before and after solid organ transplantation into routine care
Source: Front Psychol. 2025 Jan 17;15:1481643. doi: 10.3389/fpsyg.2024.1481643 (PMC11782271; doi:10.3389/fpsyg.2024.1481643)
Supplement: Supplementary file 1 [file Data_Sheet_1.PDF]

Thermo-Tx
Seelische Belastung frühzeitig erkennen und behandeln

PSYCHOLOGICAL DISTRESS AFTER SOLID ORGAN TRANSPLANTATION

1. Please circle the number (0-10) that best describes how much distress you have been experiencing in the past week including today.[1]

Extreme distress 10
9
8
7
6
5
4
3
2
1
No distress 0

2. Please indicate if any of the following has been a problem for you in the past week including today. Be sure to check YES or NO for each.

YES	NO	
		**problems in everyday life**
◯	◯	regular medical surveillance
◯	◯	daily medication schedule
◯	◯	adjustment of life style habits
◯	◯	burden of responsibility for the new organ
◯	◯	fact of never being fully well again
		**social problems**
◯	◯	feeling of being a burden to others
◯	◯	worries about family and friends
◯	◯	difficulties in talking about the transplantation
◯	◯	occupational difficulties
◯	◯	lack of support in the health care system

YES	NO	
		**worries and anxieties**
◯	◯	about the future
◯	◯	about drug side-effects
◯	◯	about infections
◯	◯	about transplant rejection and the need for a re-transplantation
		**physical and psychological problems**
◯	◯	exhaustion, mental or physical
◯	◯	sleep disorders
◯	◯	sexual problems
◯	◯	increased focus on body symptoms
◯	◯	pain
◯	◯	infections
◯	◯	drug side-effects
◯	◯	medical complications and transplant-induced illnesses

Other problems: ______________________________

3. Over the last two weeks, how often have you been bothered by the following problems?[2]

	Not at all	Several days	More than half the days	Nearly every day
Feeling nervous, anxious or on edge	◯	◯	◯	◯
Not being able to stop or control worrying	◯	◯	◯	◯
Little interest or pleasure in doing things	◯	◯	◯	◯
Feeling down, depressed, or hopeless	◯	◯	◯	◯

4. Would you like to talk to a psychologist about these problems?

◯YES ◯NO

________________ ________________________
Date Name

[1] NCCN distress thermometer (Donovan et al., 2014) [2]PHQ-4 (Kroenke et al, 2009)

Thermo-Tx
Seelische Belastung frühzeitig erkennen und behandeln

PSYCHOLOGICAL DISTRESS BEFORE SOLID ORGAN TRANSPLANTATION

1. Please circle the number (0-10) that best describes how much distress you have been experiencing in the past week including today.[1]

Extreme distress

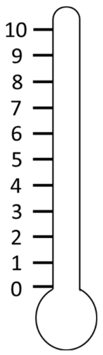

No distress

2. Please indicate if any of the following has been a problem for you in the past week including today. Be sure to check YES or NO for each.

Yes	No	**problems in everyday life**
○	○	burden of the uncertain waiting period
○	○	burden of medical treatment
○	○	fact of never being fully well again
○	○	impaired coping in everyday life
○	○	adjustment of life goals to the new situation

Yes	No	**social problems**
○	○	feeling of being a burden to others
○	○	worries about family and friends
○	○	loss of social life
○	○	social support deficits
○	○	lack of support in the health care system

Yes	No	**worries and anxieties**
○	○	about the future
○	○	about the donor organ arriving in time
○	○	about mortality
○	○	about the transplant surgery
○	○	about post-transplant medical complications

Yes	No	**physical and psychological problems**
○	○	worsening of the general health condition
○	○	sleep disorders
○	○	exhaustion, mental or physical
○	○	feelings of loss of control
○	○	sexual problems
○	○	severe physical discomforts and limitations

Other problems: ____________________

3. Over the last two weeks, how often have you been bothered by the following problems?[2]

	**Not at all**	**Several days**	**More than half the days**	**Nearly every day**
Feeling nervous, anxious or on edge	○	○	○	○
Not being able to stop or control worrying	○	○	○	○
Little interest or pleasure in doing things	○	○	○	○
Feeling down, depressed, or hopeless	○	○	○	○

4. Would you like to talk to a psychologist about these problems?

○YES ○NO

____________________ ____________________
Date Name

[1] NCCN distress thermometer (Donovan et al., 2014) [2]PHQ-4 (Kroenke et al, 2009)
